# Effects of Silicon, Chromium, and Copper on Kinetic Parameters of Precipitation during Tempering of Medium Carbon Steels

**DOI:** 10.3390/ma14061445

**Published:** 2021-03-16

**Authors:** Aleksandr Gokhman, Zbyšek Nový, Pavel Salvetr, Vasyl Ryukhtin, Pavel Strunz, Petr Motyčka, Jan Zmeko, Jakub Kotous

**Affiliations:** 1 COMTES FHT a.s., Prumyslova 995, 334 41 Dobrany, Czech Republic; aleksandr.gokhman@comtesfht.cz (A.G.); zbysek.novy@comtesfht.cz (Z.N.); petr.motycka@comtesfht.cz (P.M.); jan.zmeko@comtesfht.cz (J.Z.); jakub.kotous@comtesfht.cz (J.K.); 2 Nuclear Physics Institute, Czech Academy of Sciences, 250 68 Řež, Czech Republic; ryukhtin@ujf.cas.cz (V.R.); strunz@ujf.cas.cz (P.S.)

**Keywords:** medium carbon steels, microstructure, tempering, XRD, TEM, USANS, SANS, dilatometry, carbides, copper precipitates

## Abstract

Understanding the tempering behavior of medium carbon steels is mandatory if their mechanical properties are to be improved. For an optimal technology to be developed for this purpose, a substantial experimental basis is needed to extract quantitative information on the microstructure of the tempered material. This paper reports on the characterization of microstructural changes induced by tempering in medium-carbon steels alloyed with Si, Cr, Cu, and Mn using state-of-the-art experimental techniques. Complementarities among these techniques are highlighted. The evolution of transition carbides, cementite, and copper precipitates is described using data from X-ray diffraction, small and ultra-small angle neutron diffraction, transmission electron microscopy, and dilatometry observation. The effects of silicon, chromium, and copper on the mechanism of carbide and copper precipitation are discussed. The considerable changes found in the size and volume of copper precipitates correlate well with the difference in the yield stress between tempered steels with and without copper.

## 1. Introduction

The use of experimental techniques for characterizing the microstructure of medium-carbon alloyed with Si, Cr, Cu, and Mn steel contributes to the understanding of the kinetics of precipitation induced by heat treatment. These steels are used in a wide range of applications, e.g., as spring materials. They are characterized by an excellent combination of tensile strength, yield strength, elongation, and reduction of area [1,2,3]. Typically, their yield strength increases with tempering temperature within a specific temperature range. Reduction of area dramatically increases during tempering from the room temperature (RT) to 300 °C. Any further increase in the tempering temperature leads to a decreasing yield strength and a slow increase in the reduction of area and elongation, up to at least 400 °C [2]. Tempering after quenching has an important effect on the final properties. Tempering activates migration of carbon from supersaturated martensite. Free carbon plays a key role in each tempering stage, including carbon clustering, precipitation of ε-carbide, retained austenite decomposition, and precipitation of other tempering products such as cementite, carbides of alloying elements and others [4,5,6]. Besides carbide-based precipitates, intermetallic particles, or pure metal particles, namely copper, which form during tempering seem to be an effective means of strengthening for some types of steels. The mechanism of carbide and copper precipitation during continuous tempering was investigated in copper-bearing medium carbon steel Fe-0.44C-0.60Mn-0.21Si-0.11Cr-1.53Cu (wt%) in [7,8] by X-ray diffraction (XRD), electrical resistivity measurement, small angle neutron scattering (SANS), transmission electron microscopy (TEM), and dilatometry. However, the effects of silicon which counters segregation and the segregating chromium, which are related to the mechanism of tempering [9], have not yet been investigated using a comprehensive study comparable to [7,8]. The main goal of this paper is to report on microstructure characterization of the tempered condition in medium carbon steels with copper and without copper and with higher silicon and chromium levels than in [7,8], based on a combined study by XRD, TEM, dilatometry, SANS, and ultra-small angle neutron scattering (USANS). The materials and methods are outlined in Section 2. Section 3 is devoted to the data obtained from each individual technique. The data are compiled in Section 4 to provide a coherent picture of the tempering process and the effects of the tempering temperature and chemical composition on the mechanism of carbide and copper precipitation.

## 2. Materials and Methods

### 2.1. Materials and Heat Treatment

The material under investigation was medium carbon steel, which is widely used for making springs. Two variants were compared. The first one was 42SiCr steel and the second was 42SiCr + Cu steel, which was substantially modified by an addition of copper. Both variants were made at the company COMTES FHT a.s. Their chemical compositions are given in Table 1. These steels have higher contents of silicon and chromium than Fe-0.44C-0.75Mn-0.25Si-0.03Cr steel and Fe-0.44C-0.60Mn-0.21Si-0.11Cr-1.50Cu steel which were studied in [7,8].

The steels were melted in a vacuum induction furnace and cast into 45 kg ingots. The ingots were forged to 60 mm × 60 mm × 500 mm from the temperature of 1100 °C, then reheated to 1100 °C and hot-rolled to 16.5 mm × 94 mm × 1850 mm, which was followed by slow cooling. The highest temperature was 1100 °C. Finally, rods 13 mm in diameter and 130 mm in length were produced.

**Figure 1:**
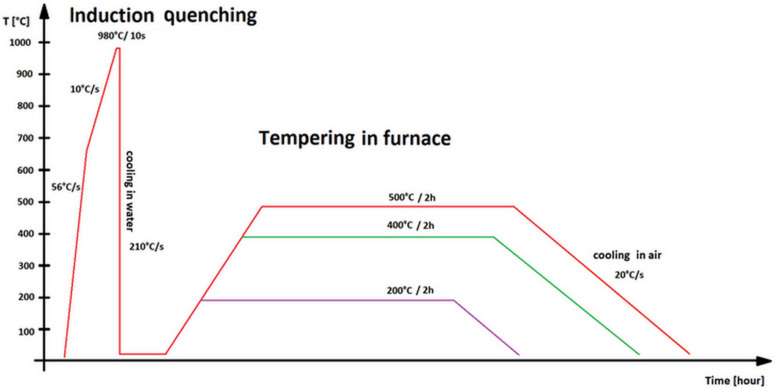
Time–temperature profile of the quenching and tempering process.

Specimens were made and induction heated to the austenitizing temperature of 980 °C for 10 s, quenched in a water bath, and then tempered at 200 °C, 400 °C, and 500 °C for two hours in an electrical chamber furnace, then air-cooled (identified below as 722, 742, and 752 for 42SiCr steel and 822, 842, and 852 for 42SiCr + Cu steel). The process of quenching and tempering is shown in Figure 1.

### 2.2. XRD Measurement

Samples for XRD measurement were prepared by polishing and etching in hydrochloric acid for 90 s to remove the surface layer formed during polishing. XRD measurements were performed using BRUKER D8 DISCOVER diffractometer (Bruker AXS GmbH, Karlsruhe, Germany) with 1.5406 Å wave length of Cu-Kα radiation. The scanning angle (2Theta) ranged from 35° to 105° with a step size of 0.15° and a measurement time per step of 0.25 s. A Pseudo-Voigt profile was used for determining the full width half maximum (FWHM) according to [10]. Then the dislocation density and microstrain were calculated according to [11].

### 2.3. TEM Observation

Metallographic samples were prepared by mechanical grinding and polishing of a surface oriented in the longitudinal direction. In order to analyze the precipitates, the samples were quenched and tempered at 200 °C, 400 °C, or 500 °C for 2 h and examined in transmission electron microscope Jeol 2100 F (JEOL, Tokyo, Japan accelerating voltage 200 kV) equipped with an energy dispersive X-ray spectrometer Oxford Instruments X-Max80 (Oxford Instruments, High Wycombe, UK) and Aztec software (Oxford Instruments, High Wycombe, UK). The types of precipitates were determined using selected area electron diffraction (SAED) and fast Fourier transform (FFT) of HRTEM images. The size of the precipitates formed during tempering was determined by NIS-elements image analysis software (AR, Laboratory Imaging, Prague, Czech Republic).

The preparation of carbon extraction replicas on Ni and Cu grids included the following steps: mechanical and electrochemical polishing, etching with 3% Nital solution, carbon deposition, etching with 10% Nital solution, and releasing the carbon replicas with particles electrolytically using a solution of 450 mL glacial acetic acid, 40 mL perchloric acid, and 10 mL distilled water using a voltage of 30 V. The carbon replicas were placed on Cu and Ni-grids. Thin foils were prepared by focused ion (Ga) beam in scanning electron microscope TESCAN LYRA3 (Tescan, Brno, Czech Republic).

### 2.4. Dilatometry

Specimens with 4 mm in diameter and 10 mm in length, which were quenched after austenitization at 980 °C for 10 s at cooling rate 100 K/s were used for the dilatometry. Then the length decrease due to precipitation was recorded using two push-rod dilatometers at continuous heating at various heating rates. The dilatometer L75PT (Linseis, Selb, Germany) with samples heated in a furnace was used for heating at lower heating rates, the quenching dilatometer L78RITA (Linseis) was used for quenching and heating at 0.1 K/s and higher heating rates. Experiments were performed under pure nitrogen, specimens were held in quartz glass push-rods with contact force of 0.3 N.

Non-isothermal tempering was performed using continuous heating with heating rates *β* = 0.003 K/s, 0.01 K/s, 0.05 K/s, 0.1 K/s, 0.5 K/s, 1 K/s, 10 K/s, and 50 K/s. The activation energy *E* and frequency factor *K_0_* for the precipitation of carbides were calculated for the two stages of tempering for 42SiCr and 42SiCr + Cu steels using an approximate expression from [12]:

(1) $$Ln\frac{\beta }{T_{i}^{2}}=-\frac{E}{RT_{i}}+Ln\frac{RK_{0}}{E}$$

Here, *R* is the gas constant, *T*_i_ indicates the inflection point in the dilatometer records obtained at various heating rates.

### 2.5. USANS and SANS Measurements

USANS data were collected at CANAM infrastructure in Nuclear Physics Institute of the Czech Academy of Sciences in Řež using a MAUD instrument [13] with several instrumental resolutions at a neutron wavelength of 2.09 Å. The samples were placed in a permanent horizontal magnetic field of about 1 T in order to minimize scattering from magnetic domains.

Pin-hole SANS measurements were performed using V4 instrument at Helmholtz Zentrum Berlin (HZB), Germany [14]. With this conventional SANS technique, one can analyze much finer microstructures (down to nm scale) than USANS, due to a larger scattering vector magnitude *Q* (*Q* = 4π sin(*θ*)/*λ*, where 2*θ* is the scattering angle, *λ* is the neutron wavelength; *λ* = 0.5 nm was used). The samples were placed in horizontal magnetic field of 2 T for the measurements. This enables nuclear and magnetic scattering contributions to be separated as shown in [15]. The data were collected using a 2D position sensitive detector. Combining several measurements with sample-to-detector distances of 1.7 m, 8 m, and 16 m, the measured SANS data covered a *Q*-range of 0.03–3 nm^−1^. The raw data were processed according to standard procedure BerSANS [16] for SANS. SASProFit software [17] was used for the USANS data fitting. It should be noted that the USANS data were measured using a slit geometry, whereas the SANS data were collected with pin-hole geometry. It is therefore impermissible to join the datasets from SANS and USANS facilities directly.

## 3. Results

### 3.1. XRD

**Figure 2:**
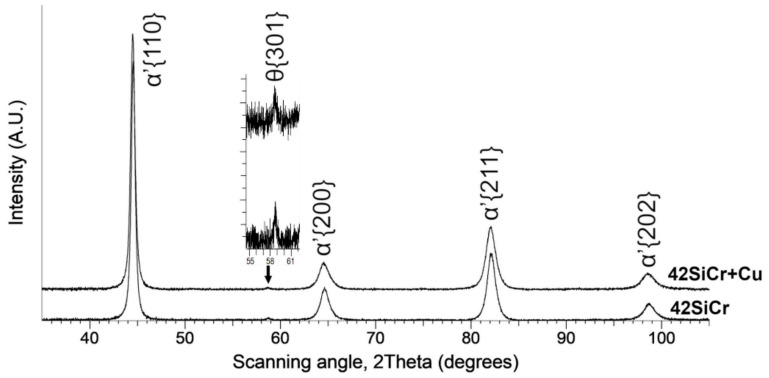
XRD patterns of 42SiCr and 42SiCr + Cu steels after quenching.

XRD patterns of quenched 42SiCr and 42SiCr + Cu steels were found to be very similar (Figure 2). Diffraction peaks of *α’* martensite were detected. A weak peak of θ cementite was found as well. No retained austenite (RA) was detected, which is in line with the relatively high martensite start (*M_s_*) temperatures: 368.4 °C for 42SiCr steel and 338.6 °C for 42SiCr + Cu steel. The *M_s_* temperatures were calculated using the following equation [18]:

(2) $$M_{s}\left({}^{\circ}C\right)=545-330C-23Mn-14C-13Ni-7Si+2Al+7Co-5Mo-13Cu\;\left(wt\%\right)$$

**Figure 3:**
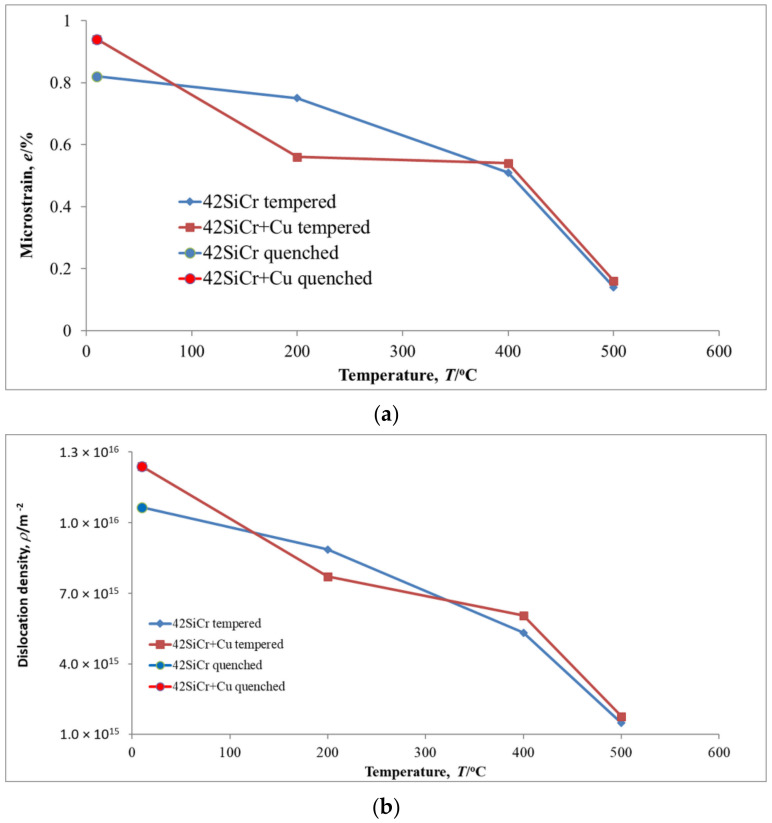
Variation in microstrain *e* (**a**) and dislocation density *ρ* (**b**) of 42SiCr and 42SiCr + Cu steels after quenching and then tempering for 2 h.

Microstrains (*e*) and dislocation density (*ρ*) of 42SiCr and 42SiCr + Cu steels after quenching and subsequent tempering, determined using XRD data in accordance with [11], are shown in Figure 3. Note that the found high value of the *ρ* in studied steels after quenching corresponds to the result [10] on the dependence of *ρ* on the silicon content. With an increase in the tempering temperature to 500 °C both *e* and *ρ* for 42SiCr and 42SiCr + Cu steels decreased significantly, i.e., almost by 7 and 6 times, respectively.

### 3.2. TEM Analysis

**Figure 4:**
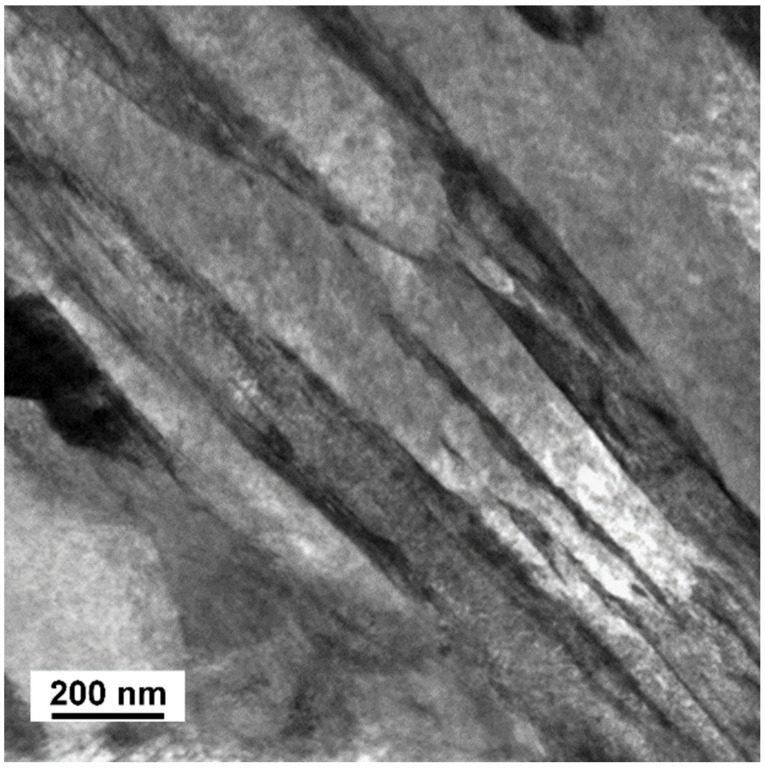
Lath martensite which formed during quenching of 42SiCr steel.

**Figure 5:**
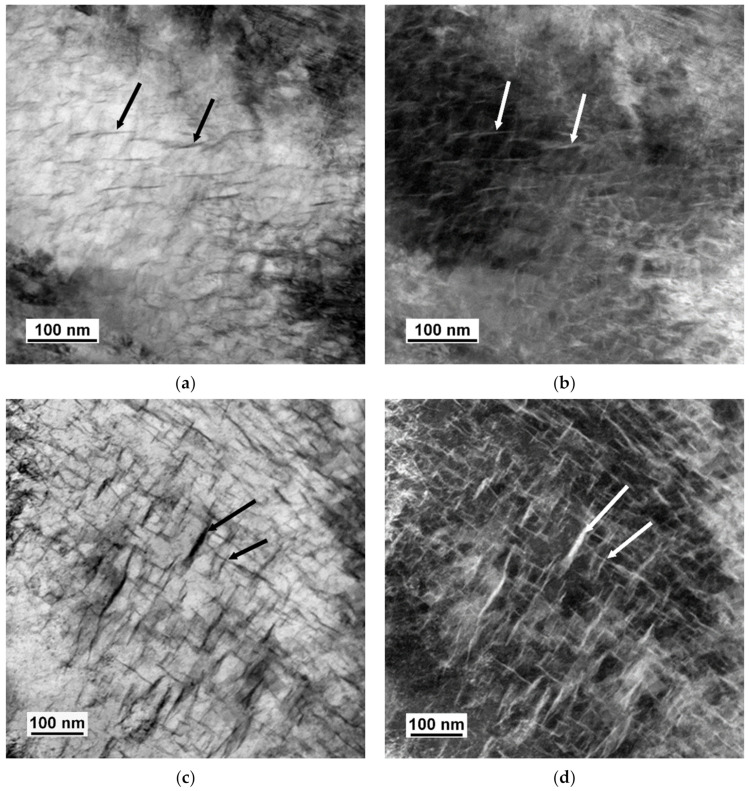
Iron transition carbides in lath martensite tempered at 200 °C for 2 h in 42SiCr steel; (**a**) bright field (BF), (**b**) dark field (DF); 42SiCr + Cu (**c**) BF, (**d**) DF.

**Figure 6:**
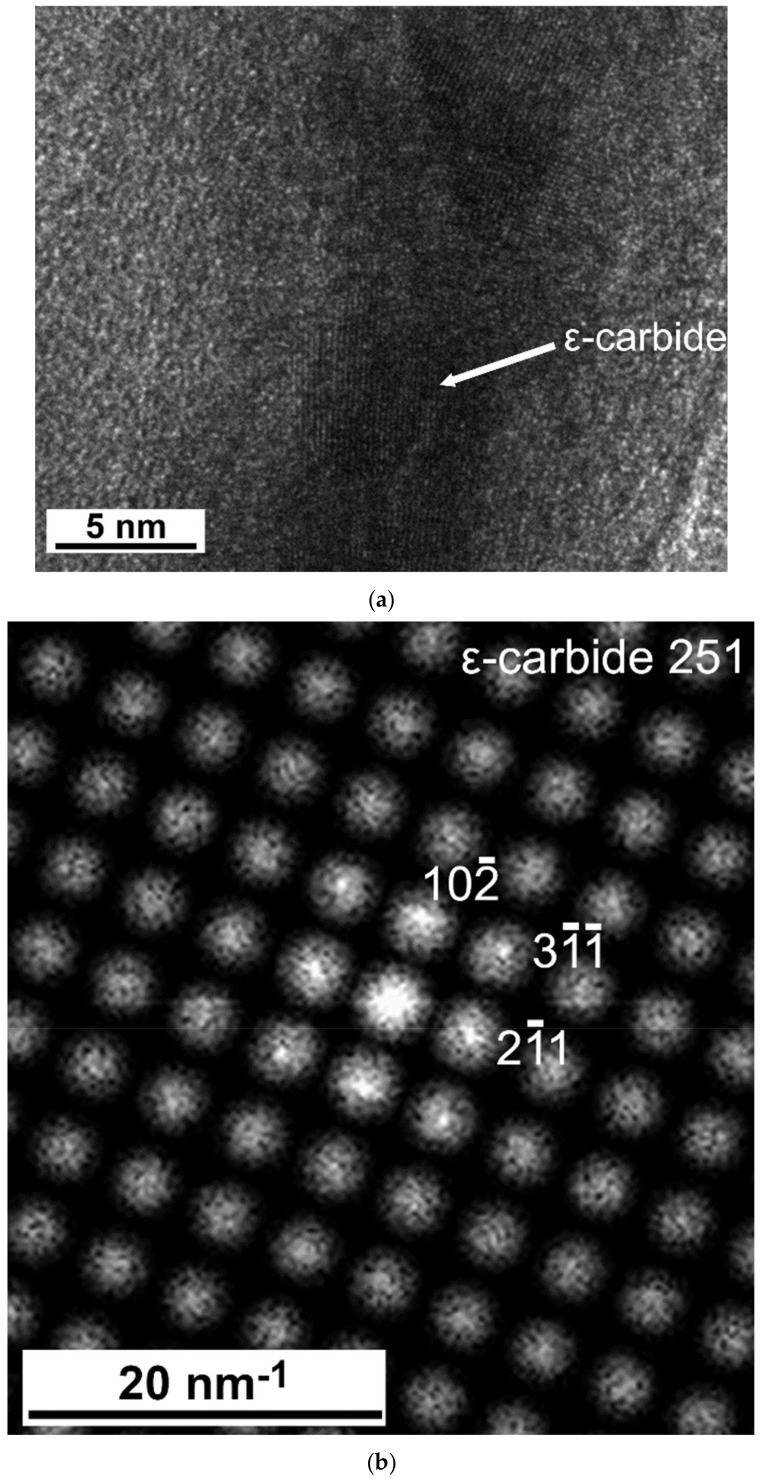
(**a**) HRTEM image of iron transition carbides in 42SiCr steel after tempering at 200 °C, (**b**) A fast Fourier transform (FFT) diagram of the micrograph in (**a**).

**Figure 7:**
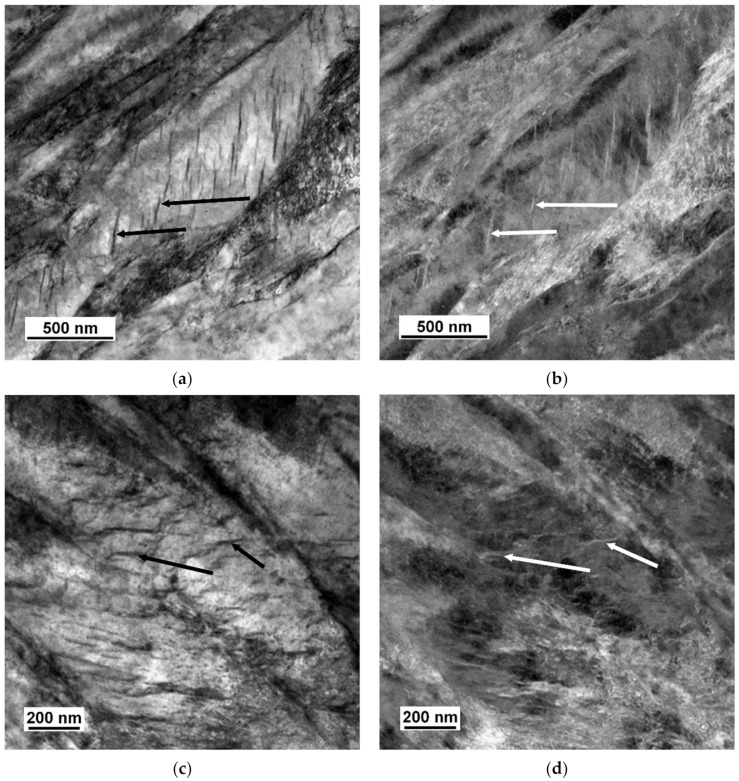
Iron carbides (indicated with black and white arrows) in 42SiCr after tempering at 400 °C (**a**) BF, (**b**) DF and in 42SiCr + Cu (**c**) BF, (**d**) DF.

**Figure 8:**
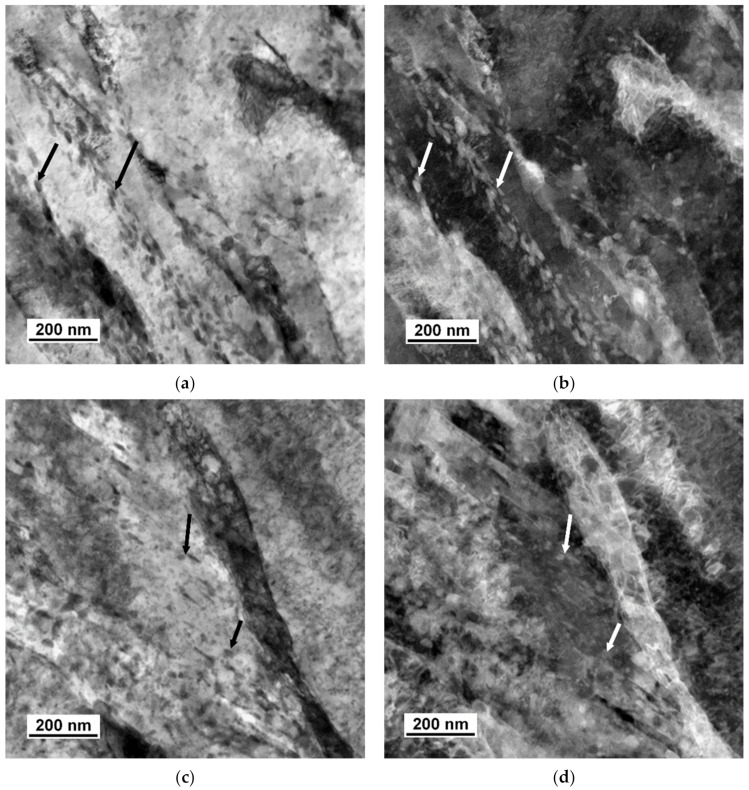
TEM micrograph showing cementite precipitates in martensite tempered at 500 °C in 42SiCr steel (**a**) BF, (**b**) DF and in 42SiCr + Cu steel (**c**) BF, (**d**) DF.

**Figure 9:**
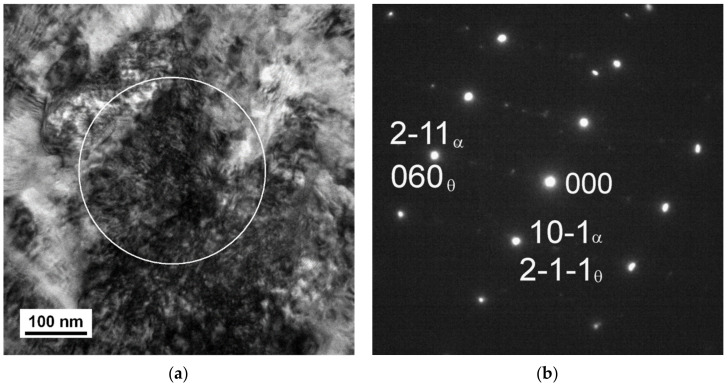
TEM micrograph of 42SiCr + Cu steel tempered at 500 °C (**a**) bright field image, (**b**) SAED pattern with zone axis [131]α. The white circle in the micrograph shows the boundary of the region for SAED analysis.

**Figure 10:**
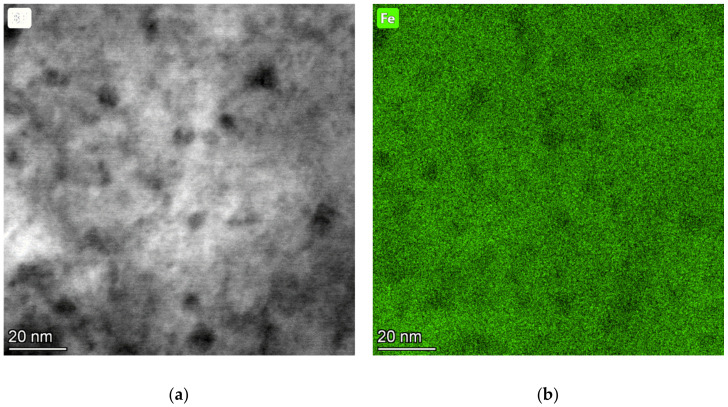

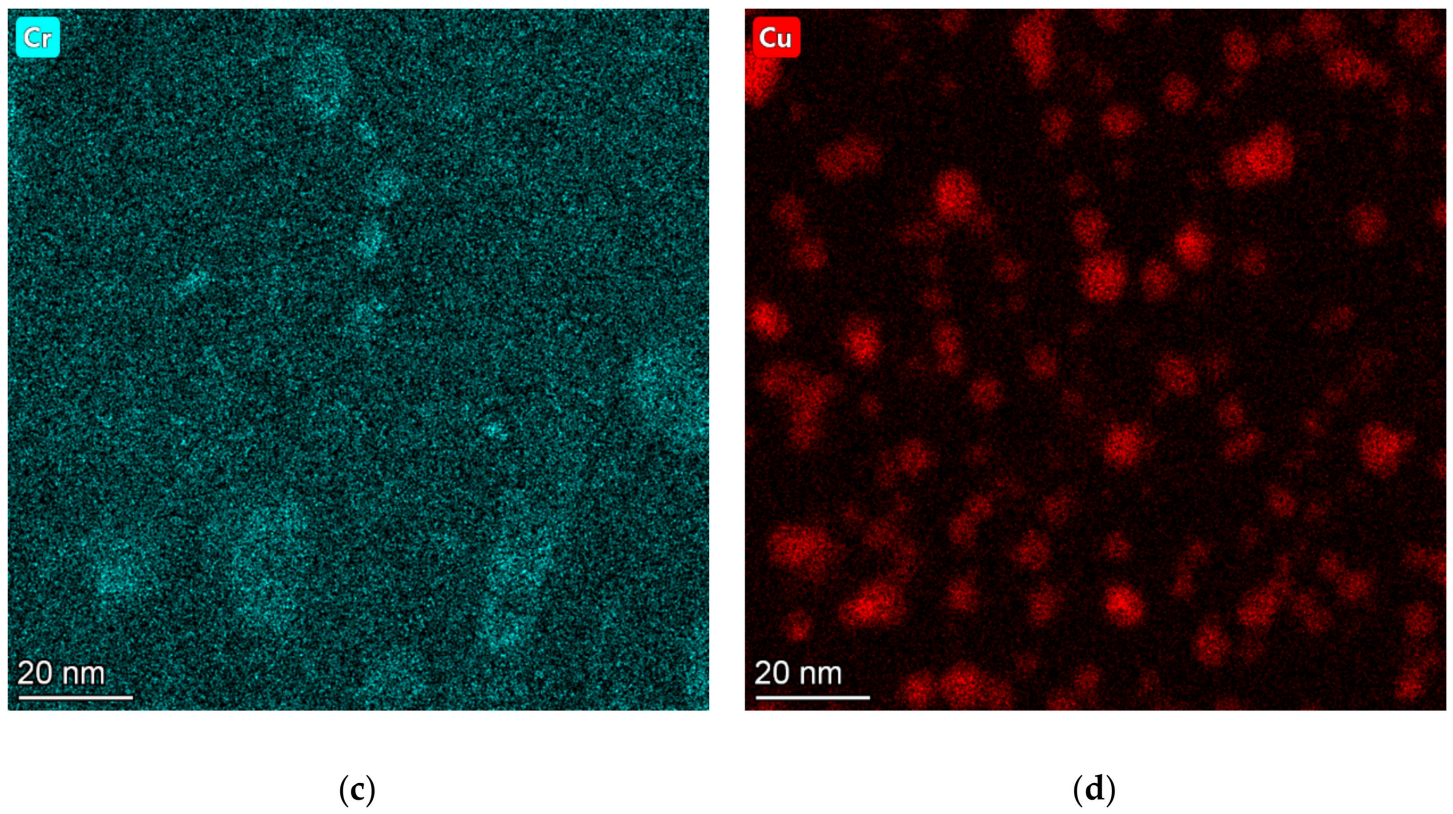
TEM-EDS map of chemical composition showing Cu-rich precipitates after tempering at 500 °C—(**a**) BF, (**b**) Fe map, (**c**) Cr map, (**d**) Cu map.

The microstructures observed in TEM after quenching and after tempering are shown in Figure 4, Figure 5, Figure 6, Figure 7, Figure 8, Figure 9 and Figure 10. There was close resemblance between as-quenched microstructures in 42SiCr and 42SiCr + Cu steels. Figure 4 is a micrograph of 42SiCr steel after quenching. Lath martensite was the dominant phase. The lath thickness ranged between 100 and 500 µm. After subsequent tempering at 200 °C for two hours, iron-transition-carbide precipitates (indicated with black and white arrows in Figure 5) were found in the interior of the martensite laths. This carbide is normally identified as ε- or η-carbide [5,19]. In this study, only ε-carbides were detected in both 722 and 822 specimens using HRTEM analysis and a fast Fourier transform (FFT). Figure 6 shows results of HRTEM-FFT analysis of the carbide particles found in 722 specimen. These precipitates of iron transition-carbides with plate-like morphology were oriented preferentially along the [010], [001] or [011] martensite direction. Similar average lengths and widths of transition ε- carbides (72.9 nm and 8.3 nm) and (72.3 nm and 7.2 nm) were found in 722 specimen and 822 specimen, respectively.

Figure 7 shows a microstructure after tempering at 400 °C for two hours, which comprises tempered martensite and particles of transitional ε-carbide. Their average length was 99 nm in specimen 742 and 125 nm in specimen 842. The width was approximately 8 nm in both specimens. After the tempering temperature had been raised to 500 °C, the treatment caused a change in the shape of the particles, as well as in their amount, diversity, and morphology. With increasing tempering temperature, the particles of transition carbides dissolved or transformed to another type. The new particles became more spherical. However, there were also rod-like or plate-like particles in the material. Coarser particles occupied the boundaries of tempered martensite, whereas the interior contained finer precipitates, see Figure 8. Using SAED, the particles were identified as cementite in a ferritic matrix (Figure 9). In Figure 9, strong diffraction spots of ferrite on the zone axis [131]α, weak diffraction spots of cementite [−102]θ, and orientation dependence [131]α-Fe//[−102]θ-Fe3C were observed. The average length of the cementite particles was 46.5 nm in specimen 752 and 48.1 nm in specimen 852. The steel modified with copper contained a considerably larger amount of very fine precipitates. The precipitates in it were distributed uniformly. Copper-rich precipitates ranging in size from 4 to 10 nm were detected, as seen in the TEM-EDS map of the chemical composition of 42SiCr + Cu steel in Figure 10. Enrichment with Cu was found exactly in the iron-depleted places.

### 3.3. Dilatometry

**Figure 11:**
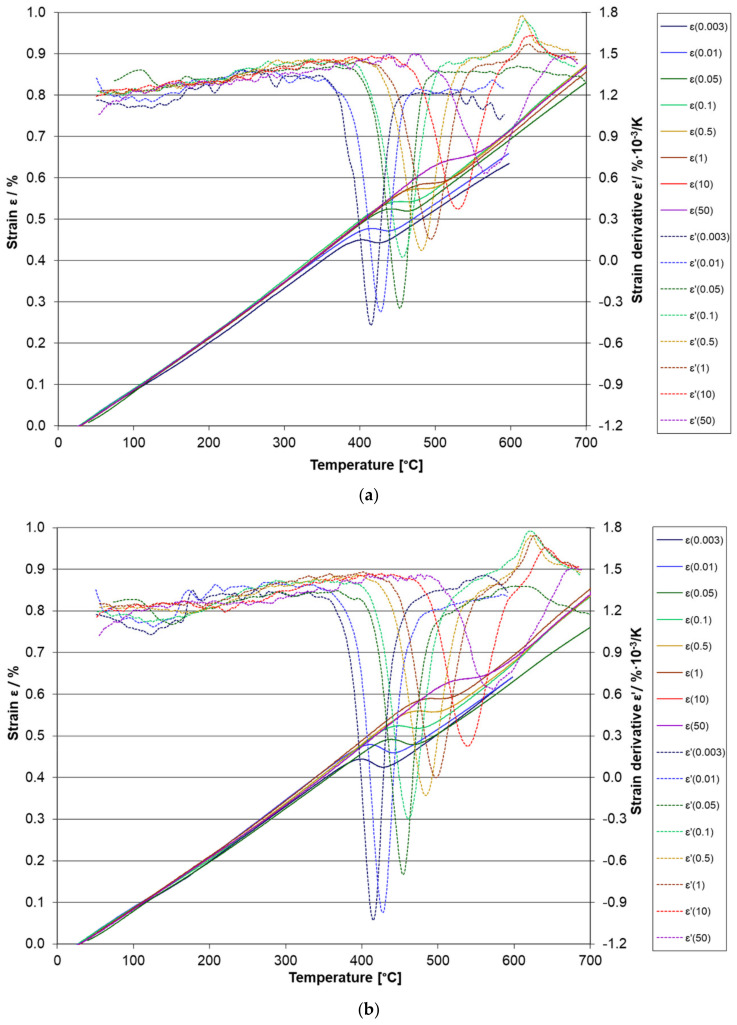
Thermal strain and its derivative for 42SiCr steel (**a**) and 42SiCr + Cu steel (**b**) tempered using heating rates *β* = 0.003 K/s, 0.01 K/s, 0.05 K/s, 0.1 K/s, 0.5 K/s, 1 K/s, 10 K/s, and 50 K/s.

**Figure 12:**
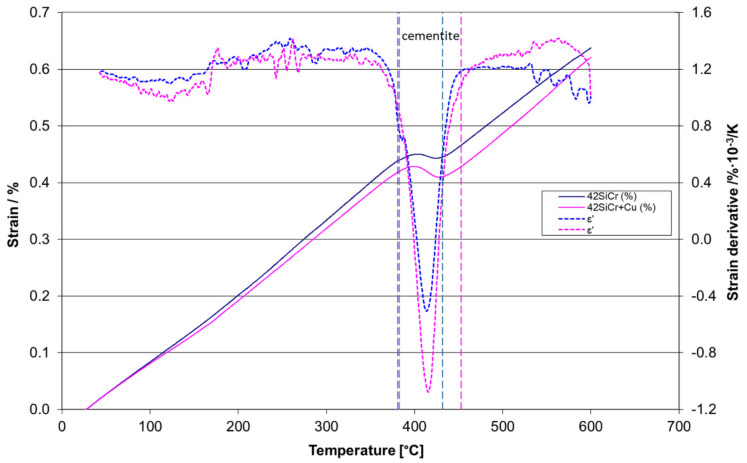
The temperature range for the formation of cementite in 42SiCr and 42SiCr + Cu steel tempered using heating rate β = 0.003 K/s.

Two contractions are found in dilatometry heating curves for 42SiCr and 42SiCr + Cu steels (Figure 11). The temperatures for inflection points (*T*_i_) of these steels and steels with low silicon and chromium levels [7,8] (*T*_i_ values for steels [7,8] were taken from Figure 3b in [8]) are presented in Table 2 and Table 3. The inflection point for the first tempering stage indicates the precipitation of ε-carbides, whereas the inflection point for the second tempering stage indicates the precipitation of cementite. A very significant effect of chemical composition was found by comparing the inflection points of cementite precipitation in the present steels and in the steels studied in [8]. Note that cementite precipitates in 42SiCr steel are between 381 °C and 432 °C and in 42SiCr + Cu steel are between 383 °C and 453 °C at a heating rate of 0.003 K/s (Figure 12). Therefore, cementite can be expected in specimens 742 and 842. Tempering peaks are larger for 42SiCr + Cu steel than for 42SiCr steel because a larger proportion of solute C atoms remain in the virgin martensite in 42SiCr + Cu steel, which might be caused by the suppressed auto-tempering during quenching [8]. The difference in peak size corresponds to the amount of strain induced by carbide precipitation. This value decreased with increasing heating rate from 0.5 K/s. Higher heating rates shift the inflection point of strain for stage II tempering to higher temperature and suppress stage I tempering. The activation energy *E* and frequency factor *K_0_* calculated for 42SiCr and 42SiCr + Cu steels and steels in [7,8] using Equation (1) are presented in Table 4 and Table 5. It was found that *E* for cementite precipitation is very sensitive to the content of silicon and chromium. There is a weak dependence of *E* for ε-carbide precipitation on Si and Cr content in the studied steels and the steels studied in [7,8]. The frequency factor for cementite precipitation was not found to be highly sensitive to chemical composition of steels. However, when it comes to precipitation of ε-carbide in steels 42SiCr and 42SiCr + Cu, it is several orders of magnitude higher in comparison to steels [7,8].

### 3.4. USANS Analysis

**Figure 13:**
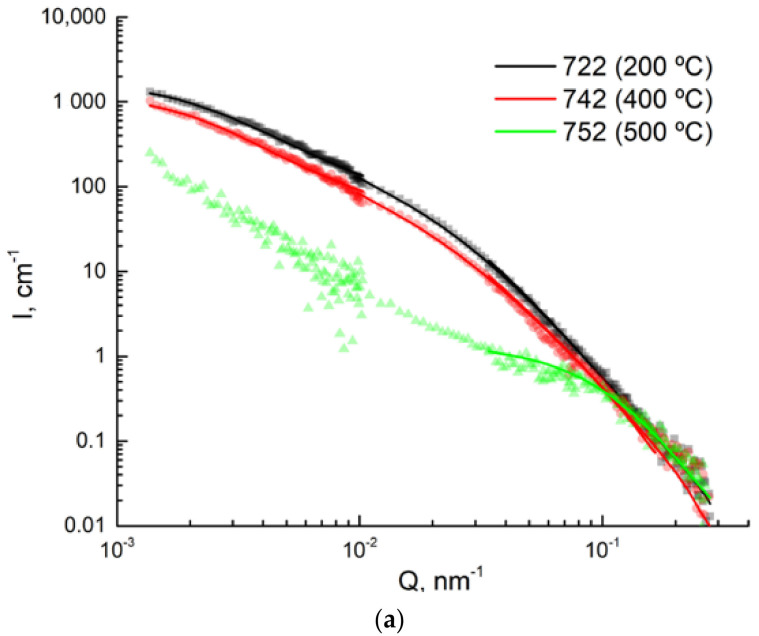

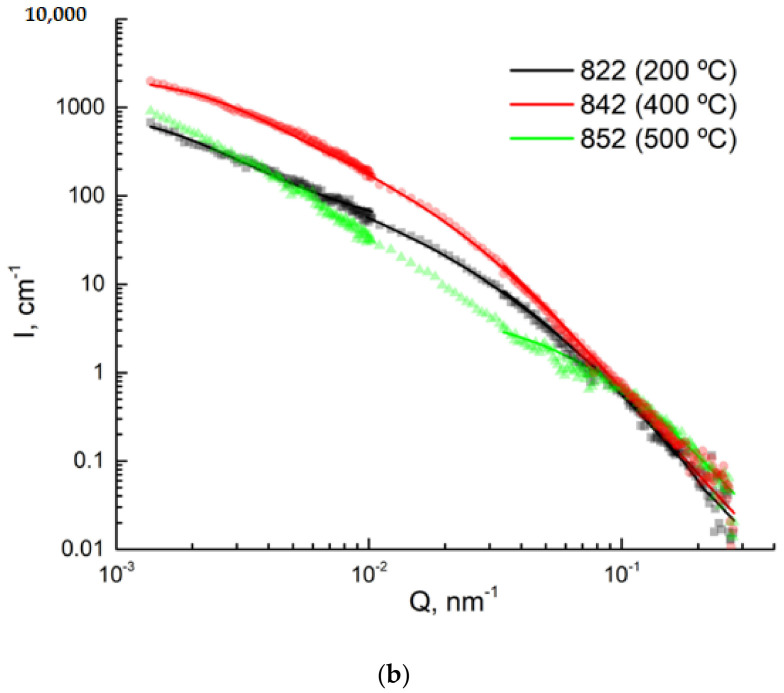
USANS data with fitted model curves: (**a**) 42SiCr, (**b**) 42SiCr + Cu.

The ex situ measured scattering curves shown in Figure 13 represent microstructural changes in the mesoscopic range after various tempering cycles followed by air cooling. The above presented TEM and dilatometry data suggest that ε-carbides generate the main contribution to the scattering from samples of both 42SiCr and 42SiCr + Cu steels after tempering for two hours at 200 °C and 400 °C (samples 722 and 742, as well as 822 and 842), although some cementite was also present in samples 742 and 842 (Figure 12). The scattering from samples tempered for two hours at 500 °C (752 and 852) occurs on cementite carbides and, in addition, on copper precipitates (in sample 852). Nevertheless, Cu precipitates are expected to be very small (see Figure 10d) and therefore do not contribute to the scattering in the USANS *Q*-range.

It is remarkable that in 42SiCr steel the scattering cross section for 400 °C-tempered sample slightly decreases when compared to the one tempered at 200 °C, whereas it is the opposite and more significant in 42SiCr + Cu steel. In 42SiCr steel heated at 500 °C, there is a significant decrease in scattering intensity across the whole *Q*-range as measured by USANS, except the largest *Q* magnitudes—which most probably indicates the presence of small cementite precipitates. The lowest *Q* magnitudes measured indicate severe inhomogeneity in the samples of 42SiCr + Cu tempered at both 400 °C and at 500 °C. It could be due to large cementite carbides or inclusions. Since the range of carbide sizes is certain to be broad, it is fully sufficient for obtaining a size estimate to fit lognormal distribution of spherical particles. The result of this fit (mean volume-weighted radius and lognormal distribution width parameter) is reported in Table 6.

**Figure 14:**
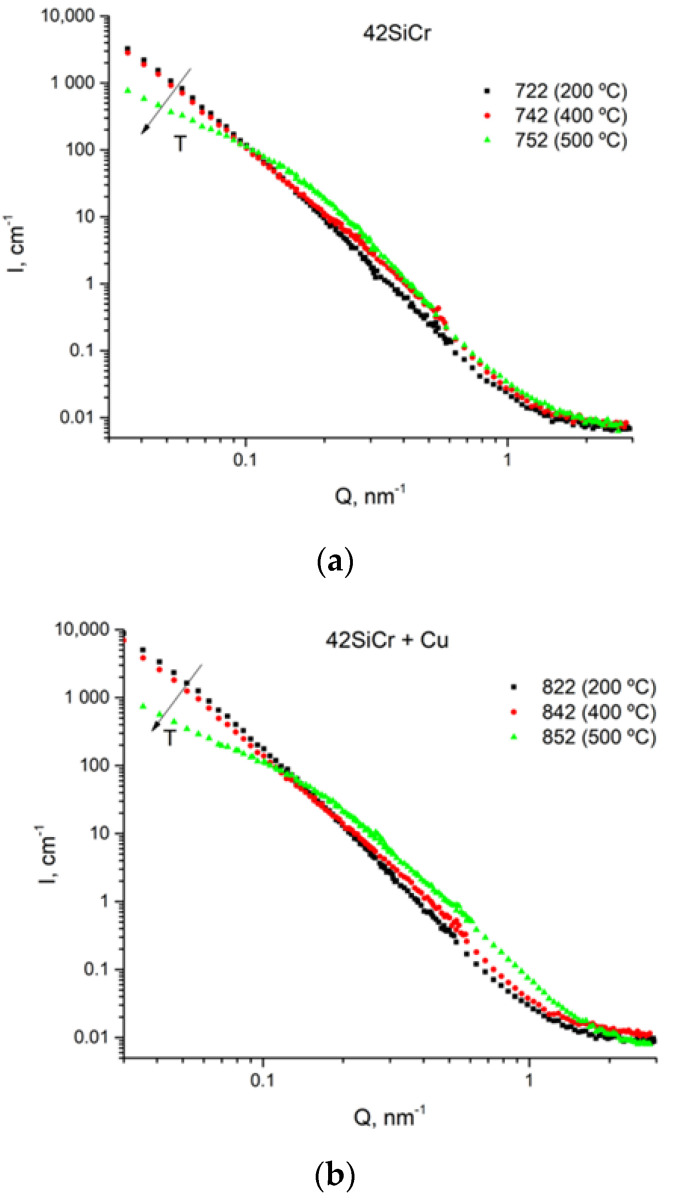
SANS curves for 42SiCr steel (**a**) and for 42SiCr + Cu steel (**b**).

**Figure 15:**
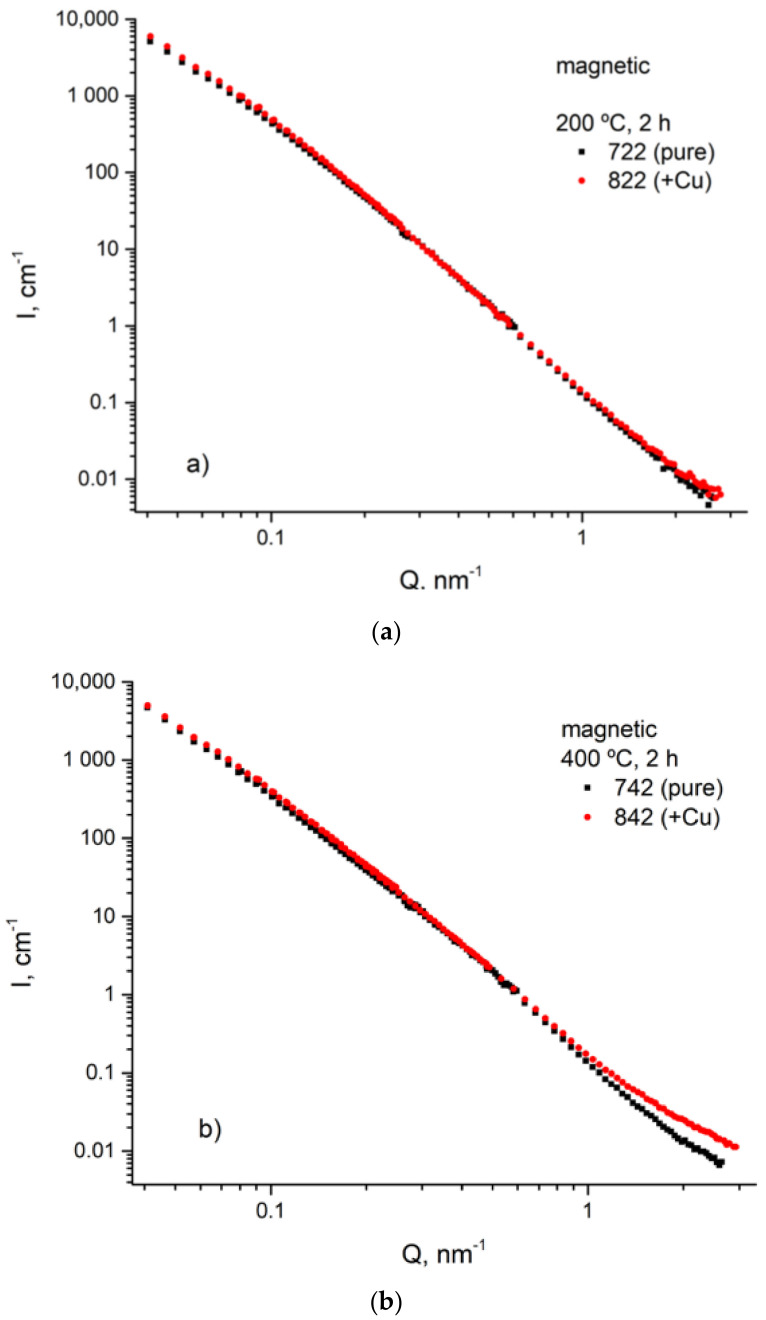

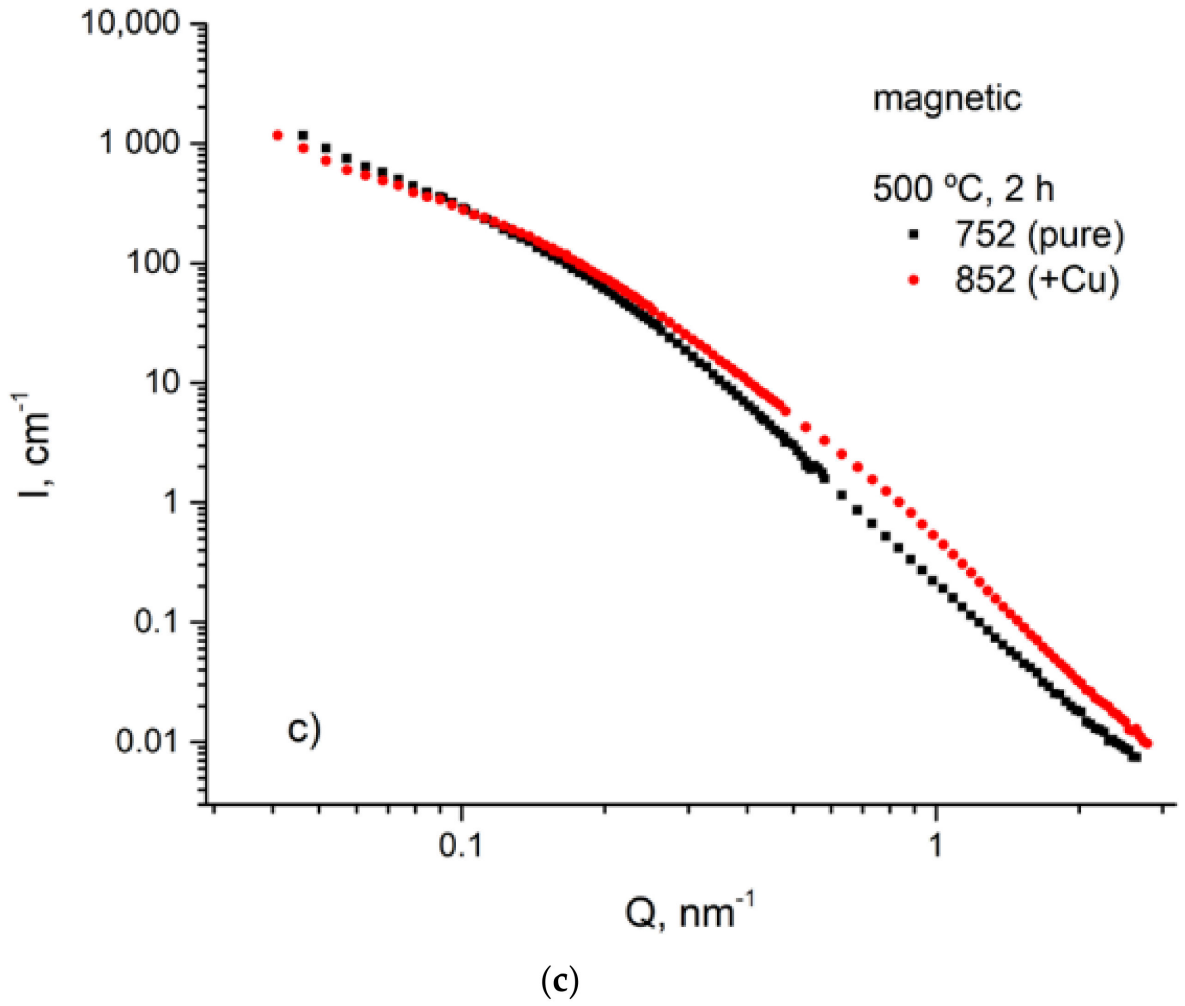
Magnetic SANS curves for samples tempered at 200 °C (**a**), 400 °C (**b**), and 500 °C (**c**) (42SiCr steel—black squares, 42SiCr + Cu steel—red circles).

The ex situ-measured SANS scattering curves are plotted in Figure 14. At first glance, the SANS curves are similar within pairs of samples (722 and 822), (742 and 842), and (752 and 852) (Figure 14). In the region of small-magnitude scattering vectors (*Q* < 0.1 nm^−1^), the scattering intensity decreases with tempering temperature in samples of the steel without copper. An opposite trend which is seen in samples with copper tempered at 400 °C and 500 °C at larger *Q* magnitude may have been caused by the occurrence of copper precipitates, according to [8,20,21]. The evolution of copper precipitates was explored by comparing the SANS magnetic scattering for samples with and without Cu tempered at the same temperatures (see Figure 15). It was assumed that the difference in the levels of silicon and chromium in 42SiCr and 42SiCr + Cu steels did not significantly affect magnetic scattering.

**Figure 16:**
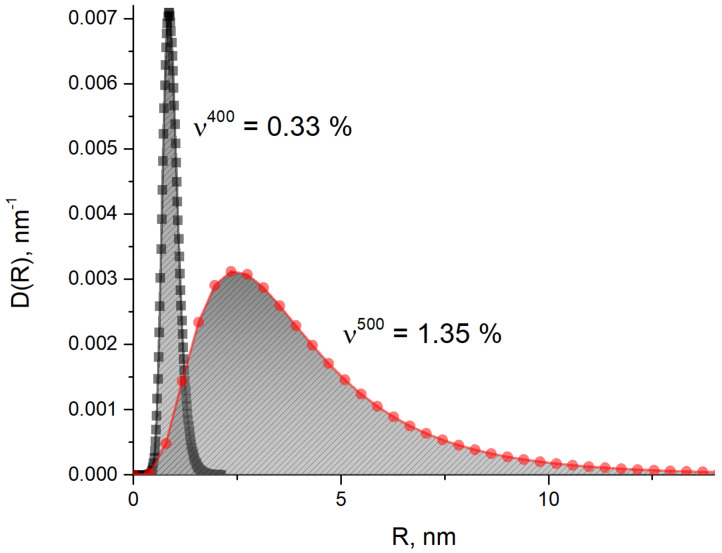
Volume weighted size distribution of Cu nanoparticles as fitted using magnetic SANS.

The differences in magnetic scattering for materials with and without Cu became apparent for higher tempering temperatures (400 °C and 500 °C). The curves were fitted using SASfit software [22]. Log-normal distributions of spherical particles were used for fitting the difference between the corresponding samples of 42SiCr and 42SiCr + Cu steels. The magnetic scattering contrast for the model was estimated by calculating the magnetic scattering length density (SLD) of iron, SLD = 5.1 · 1010 cm^−2^ because copper particles are fully nonmagnetic (SLD = 0). The fitted size distributions of spherical particles weighted by volume are shown in Figure 16. The integrals over the size distributions provide the total volume fractions of copper precipitates: v^400^ = 0.33% for the sample tempered at 400 °C (sample 842) and ν^500^ = 1.35% for 500 °C (sample 852), respectively. The resulting values may be slightly lower than the actual ones since they were close to the instrumental resolution limit, especially with sample 842. The mean radii of nanoparticles <R>*_V_* = 0.8 nm for sample 842 and <R>*_V_* = 4.1 nm for sample 852 were found from the fitted volume weighted size distributions (Table 7) [23].

## 4. Discussion

### 4.1. Phase and Microstructure Analysis

The phase compositions of 42SiCr and 42SiCr + Cu steels were determined using data from XRD, TEM, SANS, and dilatometry. Lath martensite was the dominant phase in both steels after quenching, according to TEM data. In addition, XRD analysis showed that some cementite was present in both steels even after quenching, which was due to what is referred to as auto-tempering. No retained austenite was found by XRD, which is in agreement with the relatively high martensite start temperatures: 368.4 °C for 42SiCr steel and 338.6 °C for 42SiCr + Cu steel. According to TEM observation and dilatometry, ε–carbides formed in 42SiCr and 42SiCr + Cu steels as a result of isothermal tempering at 200°C and 400 °C, as well as non-isothermal tempering with temperatures of inflection points in the range of 121–145 °C. The latter is similar to the data obtained for the steels in [7,8]. Cementite particles formed in 42SiCr and 42SiCr + Cu steels as a result of non-isothermal tempering with temperatures of inflection points in the range of 414–574 °C, whereas a range of 300–416 °C was found in the steels in [7,8]. Therefore, it can be argued that the addition of Si slightly alters the kinetics of formation of ε-carbides but significantly affects the kinetics of cementite formation in 42SiCr and 42SiCr + Cu steels, in which the Si content is almost 2 wt% higher than in steels in [7,8]. The physical basis of this impact of silicon is the formation of a Si-rich layer that slows down the growth of cementite outside in the iron matrix [9]. The segregation effect associated with the rejection of Cr atoms into cementite [9] was obviously weaker than the Si effects in the present steels, although the Cr content in 42SiCr and 42SiCr + Cu is about 1 wt% higher than in steels reported in [7,8]. The microstructure of 42SiCr and 42SiCr + Cu after isothermal tempering at 500 °C contains cementite particles in *α*-iron matrix. In addition, there are Cu-precipitates in 42SiCr + Cu steel, according to TEM-EDS and SANS data. The tempering peaks are larger for 42SiCr + Cu steel than for 42SiCr steel, which indicates that the amount of strain caused by carbide precipitation depends on the Cu content. Cu atoms suppressed auto-tempering during quenching and thus caused more carbon atoms to remain in virgin martensite. Isothermal tempering at 400 °C leads to the formation of cementite particles in both 42SiCr and 42SiCr + Cu steels, according to dilatometry data, as well as to formation of Cu precipitates in 42SiCr + Cu steel, according to SANS data. Note that Cu precipitation was not found in steels [7,8] for tempering at 400 °C.

### 4.2. Activation Energy of Carbides

The activation energies for transformation of carbides were calculated from dilatometry data, i.e., temperatures of inflection points for a set of heating rates using Equation (1). The activation energy for precipitation of ε-carbides found for the 42SiCr steel (125 kJ/mol) is similar to that for the Cu-free steel [7,8] and lies between the activation energy for the volume diffusion of C atoms (80 kJ/mol) in the martensitic matrix and the value of activation energy for the diffusion of Fe atoms (134 kJ/mol) along the dislocation lines in the matrix of bcc iron [24]. The activation energy for precipitation of ε-carbides in the 42SiCr + Cu steel was 136 kJ/mol which is higher than for the Cu–bearing steel in [7,8] (111 kJ/mol). The activation energies for the precipitation of cementite particles in 42SiCr and 42SiCr + Cu steels (288 kJ/mol and 280 kJ/mol) exceed those for steels [7,8] (206 kJ/mol and 225 kJ/mol), but are not very far from the activation energy for volume diffusion of Fe in bcc iron (251 kJ/mol) [25]. The above-mentioned increase in the activation energy may be caused by the effect of Si on the activation energy for self-diffusivity of Fe in the matrix of the steels studied in a manner similar to that found in [26].

### 4.3. Mechanism of Epsilon-Carbide and Cementite Formation

The activation energies found above suggest that the mechanism of carbide precipitation in the steels under investigation is similar to that in steels [7,8] in [8,12]: the diffusion of Fe atoms along the dislocation lines controls the reaction during the formation of ε-carbides in both 42SiCr and 42SiCr + Cu steels, whereas cementite particles form on the dislocation lines due to the dissolution ε-carbides and then their growth is controlled by the volume diffusion of Fe atoms. Thus, it can be concluded that a simultaneous increase in the content of the anti-segregating element Si from 0.21 wt% up to 2.40 wt%, the segregating element Cr from 0.03wt% up to 1.34wt% and Cu from 0 wt% to 1.5 wt%, with a decrease in C level from 0.44 to 0.40 wt% does not qualitatively change the mechanism of carbide formation induced by tempering. There is an opposite change in the USANS scattering cross section with the tempering temperature increasing from 200 °C to 400 °C in 42SiCr and 42SiCr + Cu steels (Figure 13). The reason may be the effect of Cu described in [8] where a larger amount of solute C atoms remain in the virgin martensite of 42SiCr + Cu steel, which could be caused by the suppressed auto-tempering during quenching. The latter leads to additional precipitation of carbides in 42SiCr + Cu steel in comparison with 42SiCr steel.

### 4.4. Size and Morphology of Carbides

The size and morphology of carbides were determined using TEM and USANS. According to TEM data, ε-carbides exhibit a similar mean length of about 70 nm and similar mean width of about 7.7 nm in both 42SiCr and 42SiCr + Cu steels after isothermal tempering at 200 °C for two hours. The mean length increases up to 99 nm and to 125 nm in 42SiCr and 42SiCr + Cu steel, respectively, when tempered at 400 °C for two hours but the mean width turns out to be almost identical with isothermal tempering at 200 °C. In these samples, ε-carbides resemble thin plates oriented perpendicular to the plane of observation. The shape of cementite particles was found to be more complex. They were rod-like, plate-like, or oval particles. Their mean size was smaller than that of ε-carbides. It was about of 47 nm for 42SiCr and 42SiCr + Cu steels tempered at temperature 500 °C for two hours. The mean size (double the mean radius) of carbides is larger from USANS data than from TEM. It should be noted that USANS yields a volume weighted mean value whereas TEM provides number weighted mean size. For the size distribution as broad as this (see Table 6), this difference is thus fully understandable. The fit of the scattering from ε-carbides leads to an estimate of the mean size (volume weighted) of these carbides, which is in a reasonable agreement with the number weighted size distribution from TEM image processing. Both TEM and USANS found a similar trend in the mean carbide size with an increase in the tempering temperature from 400 °C to 500 °C and close mean sizes of these carbides in FeCr and FeCr + Cu steels tempered at 500 °C. USANS can thus be recommended for characterizing carbides of mesoscopic size in these steels in case difficulties occur using TEM, for example, when examining microstructures after severe cold deformation. The data on the size of cementite particles can be useful for calculating the cementite precipitation strengthening component according to [10].

### 4.5. Size and Morphology of Cu Particles

The size and morphology of Cu particles were studied using SANS and TEM-EDS. Data were obtained on copper precipitates in 42SiCr + Cu steel tempered at 500 °C for two hours. The precipitates have an average radius of 4.1 nm according to SANS and 3.7 nm according to volume weighted size distribution functions calculated from the number distribution function $D_{N}\left(R\right):\;D_{V}\left(R\right)\;=D_{N}\left(R\right)\times \frac{4\pi }{3}R^{3}$, where $D_{N}\left(R\right)$ is calculated based on the TEM-EDS data presented in Figure 10. Differences in magnetic scattering between 42SiCr and 42SiCr + Cu steels were not observed for the temperature of 200 °C, but they emerged at higher tempering temperatures (400 °C and 500 °C). Copper precipitation was reported after tempering between 400 °C and 600 °C [27]. This confirms the assumption about the insignificant impact of the difference between silicon and chromium contents in 42SiCr and 42SiCr + Cu steels on the magnetic part of SANS. The SANS experiment complements the TEM-EDS measurements, which have a low detection limit for bcc iron (about 1.5 nm). Small copper precipitates that are invisible in TEM-EDS, with a mean radius of 0.8 nm in sample 842, were detected by SANS. After 2-h tempering at 400 °C, only partial nucleation of copper precipitates occurs (a fitted volume fraction is 0.33%, i.e., about 1/5 of the amount corresponding to the nominal Cu content). The mean size of the copper particles becomes five times larger (<R> = 4.1 nm) for T = 500 °C, and almost all Cu atoms enter the precipitates (the volume fraction resulting from the fit to the SANS data is 1.35%). Note that this value of the volume fraction was between the results obtained in [7] using SANS (1.6 wt%) and the value from measurement of electrical resistivity (1.0 wt%) for the steel in [7,8] with Cu, which was isothermally tempered at 500 °C for 10,000 s (2.8 h). The mean size of copper particles found in our study was also close to that for the steel in [7,8] isothermally tempered at 500 °C for 5700 s. The A-ratio = *I_mag_/I_nuc_*+1 calculated from the magnetic and nuclear scattering intensities (*I_mag_* and *I_nuc_*) shows that the copper precipitates in the samples did not contain just copper. The averaged A-ratio was found to be 5.18 and 5.43 for samples 842 and 852, respectively. This means that the copper precipitates in these samples were enriched in copper, but they were not pure copper precipitates (A-ratio would be equal to 1.4 for vacancy clusters, whereas it would be equal to 8.29 for pure copper precipitates [28]). Our statement about the multiple components in the copper precipitates in the present materials is consistent with the data from atom-probe tomography [29] and the results of calculation using the classical theory of nucleation [30].

### 4.6. Yield Stress vs. Cu Precipitation

**Figure 17:**
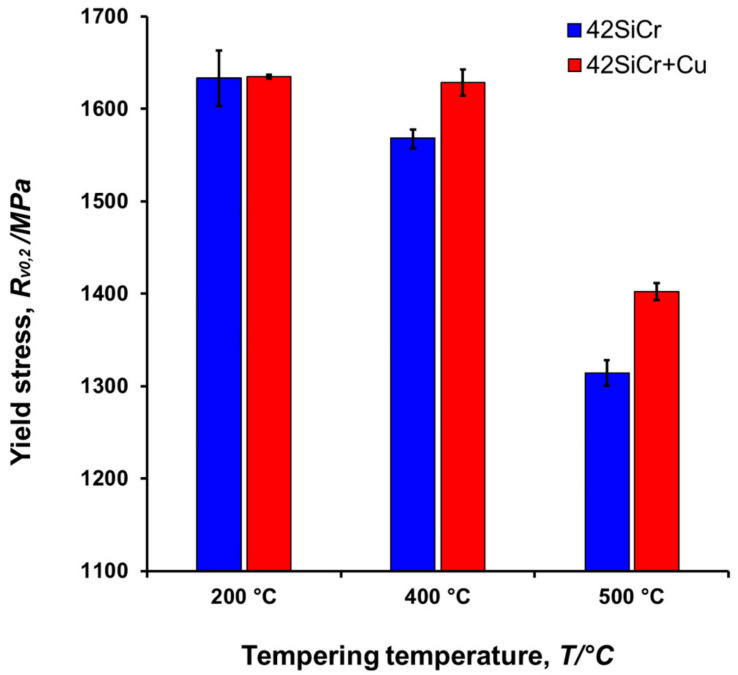
Yield stress *R_Vp_*_0,2_ of steels 42SiCr and 42SiCr + Cu tempered for two hours.

The above-mentioned steep change in the size and volume fraction of copper particles correlates well with the change in yield stress, *R_Vp_*_0,2_ of 42SiCr and 42SiCr + Cu steels tempered at elevated temperatures (Figure 17) [31].

The change in yield stress ∆*R_Vp0,2_* can be estimated using the Russel and Brown model [32]:

(3) $$\Delta R_{vp0.2}=MG_{2}\left(b/L\right){\left\{1-{\left(\frac{E_{1}}{E_{2}}\right)}^{2}\right\}}^{\frac{3}{4}}$$

where

(4) $$\frac{E_{1}}{E_{2}}=\frac{G_{1}}{G_{2}}\frac{log\left(\frac{R}{r_{0}}\right)}{log\left(\frac{R_{0}}{r_{0}}\right)}+\frac{log\left(\frac{R_{0}}{R}\right)}{log\left(\frac{R_{0}}{r_{0}}\right)},$$

In Equations (3) and (4), *M* is the Taylor factor (=3.05), *G_1_* is the precipitate shear modulus, *G_2_* is the matrix shear modulus (=83 GPa), *b* is the Burger’s vector (=0.248 nm), *r_0_* is the inner cutoff radius of dislocation *r_0_* (=2.5 b), *R_0_* is the outer cutoff radius of dislocation (*R_0_* = 2500 b), *R* is the radius of the precipitate (*R* = 0.79 nm and 4.1 nm for samples 842 and 852), *L* is the inter-precipitate spacing, which is given by $L=1.77\frac{R}{\sqrt{v}}$, and *v* is the volume fraction of precipitates (*v* =0.0033 and 0.0135 for samples 842 and 852) (*L* = 24.7 nm and 62.5 nm for samples 842 and 852). According to [7], the chemical composition of copper precipitates changes with tempering time from a multicomponent type to almost pure copper. Therefore, the value of *G_1_* is not always equal to 49 GPa which characterizes pure copper [32]. The values of *G_1_* = 74.7 GPa and 76.8 GPa lead to calculated data from Equations (3) and (4) which are close to the experimental values Δ*R_Vp0,2_* = 61 MPa and 89 MPa for samples (742, 842) and (752, 852), respectively. A similar deviation of the shear modulus of copper precipitates from the shear modulus for pure copper was found in [33] (*G*_1_ = 78.0 GPa).

### 4.7. General Microstructure Features

Many cementite particles of various shapes occurred in both 42SiCr and 42SiCr + Cu steels during tempering between 200 and 500 °C. At the same time, the microstructure of the matrix changed dramatically. The dislocation density (*ρ*) and microstrain (*e*) for 42SiCr and 42SiCr + Cu steels decreased, according to XRD, almost by 7 and 6 times, respectively, due to tempering in this temperature range, which produced recovered ferrite.

## 5. Conclusions

The influence of a simultaneous increase in Si and Cr and Cu contents on precipitation kinetics in medium carbon steels was investigated using XRD, TEM, USANS, SANS, and dilatometry. The results obtained for 42SiCr steel (Fe-0.39C-0.65Mn-2.02Si-1.21Cr-0.09Cu) and 42SiCr + Cu steel (Fe-0.41C-0.65Mn-2.40Si-1.34Cr-1.50Cu) were compared with those published for steel (Fe-0.44C-0.75Mn-0.25Si-0.03Cr) and steel (Fe-0.44C-0.60Mn-0.21Si-0.11Cr-1.50Cu) which contain similar C, Mn, and Cu at similar levels as in the steels studied, but possess a lower content of Si and Cr. Several new findings on precipitation kinetics were gathered:

1. An increase in the Si content from 0.03 wt% to 2.40 wt% leads to the significant increase (by 80 kJ/mol) of the activation energy for cementite formation and slight increase of it for ε-carbide formation. The mechanism of copper precipitation is insensitive to Si and Cr increase. On the other hand, Cu addition led to an increase in the strain present in the material. However, this effect decreases with non-isothermal tempering using continuous heating with an increase in heating rate from 0.5K/s.
2. SANS and USANS were used as complementary methods to electron imaging. Utilizing of magnetic SANS was especially efficient since it provided of size and volume parameters of the fine Cu precipitations.
3. The found steep change in the size and volume fraction of copper particles correlates well with the difference in yield strength, *R_Vp_*_0,2_ between 42SiCr and 42SiCr + Cu steels tempered at elevated temperatures. This effect can be described using the Russel-Brown model.
4. The copper precipitates in 42SiCr + Cu steel tempered at 400 °C and 500 °C are enriched in copper, but they are not pure copper particles.
5. Many cementite carbides of various shapes formed in both 42SiCr and 42SiCr + Cu steels during tempering between 200 and 500 °C. At the same time, the microstructure of the matrix changed dramatically. The dislocation density and microstrain for 42SiCr and 42SiCr + Cu steels decreased almost 7 to 6 times, respectively, due to tempering in this temperature range, which produced recovered ferrite.

## Figures and Tables

**Table 1:** Chemical composition of medium carbon steels in the present study (wt%).

Steel	C	Si	Mn	P	S	Cr	Mo	Ni	Cu	Ti	V	Fe
42SiCr	0.39	2.02	0.65	0.011	0.003	1.21	0.03	0.07	0.09	0.004	0.012	bal.
42SiCr + Cu	0.41	2.40	0.65	0.011	0.003	1.34	0.03	0.07	1.50	0.004	0.012	bal.

**Table 2:** Temperatures corresponding to inflection points (*T*_i_) at different heating rates (*β*) for stage I of tempering in the dilatometry data.

*β* (K/s)	42SiCr	42SiCr + Cu	Steel without Cu [7,8]	Steel with Cu [7,8]
0.003	126 °C	125 °C	-	-
0.01	121 °C	125 °C	-	-
0.05	142 °C	145 °C	-	-
0.1	no observed	131 °C	151 °C	144 °C

**Table 3:** Temperatures corresponding to inflection points (*T*_i_) at different heating rates (*β*) for stage II of tempering in the dilatometry data.

*β* (K/s)	42SiCr	42SiCr + Cu	Steel without Cu [7,8]	Steel with Cu [7,8]
0.003	414 °C	414 °C	-	-
0.01	426 °C	427 °C	-	-
0.05	452 °C	453 °C	-	-
0.1	456 °C	461 °C	300 °C	337 °C
0.5	481 °C	484 °C	325 °C	345 °C
1	493 °C	497 °C	328 °C	352 °C
10	529 °C	539 °C	355 °C	391 °C
50	571 °C	574 °C	390 °C	416 °C

**Table 4:** Activation energy *E* calculated for 42SiCr and 42SiCr + Cu steels and its comparison with steels without and with Cu [7,8], using Equation (1).

Stage	Activation Energy, *E* (kJ/mol) 42SiCr Steel	Activation Energy, *E* (kJ/mol) 42SiCr + Cu Steel	Activation Energy, *E* (kJ/mol) Steel without Cu [7,8]	Activation Energy, *E* (kJ/mol) Steel with Cu [7,8]
I	125	136	120	111
II	288	280	206	225

**Table 5:** Parameter *K*_0_ calculated for 42SiCr and 42SiCr + Cu steels and its comparison with steels without and with Cu [7,8], using Equation (1).

Stage	Frequency Factor, *K*_0_ (s^−1^) 42SiCr Steel	Frequency Factor, *K*_0_ (s^−1^) 42SiCr + Cu Steel	Frequency Factor, *K*_0_ (s^−1^) Steel without Cu [8]	Frequency Factor, *K*_0_(s^−1^) Steel with Cu [8]
I	1.78 × 10^13^	7.18 × 10^14^	4.85 × 10^10^	9.11 × 10^12^
II	2.25 × 10^18^	4.79 × 10^17^	3.60 × 10^17^	6.11 × 10^18^

**Table 6:** Model parameters obtained by fitting the USANS data. <R> is the mean radius and σ is the width of the lognormal distribution.

Sample No.	Sample Type	Tempering Temperature	<R>, nm	σ
722	42SiCr	200 °C	109 ± 3	0.71
742	42SiCr	400 °C	108 ± 3	0.86
752	42SiCr	500 °C	18.8 ± 1.1	0.42
822	42SiCr + Cu	200 °C	82 ± 3	0.78
842	42SiCr + Cu	400 °C	117 ± 4	0.70
852	42SiCr + Cu	500 °C	23.1 ± 1.4	0.63

**Table 7:** Fitted parameters of magnetic scattering.

	*T*, °C	*µ*, nm	*σ*, nm	<*R*>_V_, nm	*ν*, %
842–742	400	0.78	0.21	0.79	0.33
852–752	500	1.22	0.59	4.10	1.35

## Data Availability

The data presented in this study are available on request from the corresponding author.
